# Simulation and optimization of the impacts of metal-organic frameworks on the hydrogen adsorption using computational fluid dynamics and artificial neural networks

**DOI:** 10.1038/s41598-023-45391-x

**Published:** 2023-10-21

**Authors:** Hossein Pourrahmani, Mohammad Hadi Mohammadi, Bahar Pourhasani, Ayat Gharehghani, Mahdi Moghimi, Jan Van herle

**Affiliations:** 1 Group of Energy Materials, École Polytechnique Fédérale de Lausanne, Sion, 1951 Switzerland; 2 School of Mechanical Engineering, Iran University of Science and Technology (IUST), Tehran, Iran; 3 Graduate university of advanced technology, Kerman, Iran

**Keywords:** Hydrogen storage, Mechanical engineering, Chemical engineering

## Abstract

One of the barriers to further commercialization of the proton exchange membrane fuel cell (PEMFC) is hydrogen storage. Conventional methods are based on pressurizing the hydrogen up to 700 bar. The focus of this study is to characterize the hydrogen storage capacity of hydrogen tanks filled with MOF-5 at low pressures. Thus, Computational Fluid Dynamic (CFD) was used in a transient condition to analyze the hydrogen storage. Benefiting from the CFD model, three input parameters of the MOF-5, namely, density, specific heat, and conductivity, were utilized to develop an artificial neural network (ANN) model to find the highest mass of adsorption at the lowest required pressure. The optimum possible MOF among 729220 different possibilities, which enables the adsorption of 0.0099 kg at 139 bar, was found using a newly defined parameter called Pressure Adsorption Parameter (PAP).

## Introduction

The existing barriers toward the usage of fossil fuels as the prime mover of the cars have improved the commercialization of the fuel cells and batteries to replace the internal combustion engines (ICEs)^1^. Although the required infrastructure is already established for the ICE cars, the low number of hydrogen refueling stations^2^, low range of batteries^3^, high charging time of the batteries^4^, and the size/weight of the hydrogen tanks^5^ are the main concerns toward the transition from ICE cars to environmentally friendly alternatives.

Fuel cells can be directly used in the vehicles as the prime mover (mobility applications)^6^, or they can be considered as the energy provider of the electric vehicle charging stations (stationary applications)^7^. Although hydrogen storage is not considered as an obstacle for stationary applications, the required weight and size of the hydrogen tanks are barriers to facilitating the usage of hydrogen in the automotive sector^8^. Based on the given standards^9^, it is possible to pressurize hydrogen up to 700 bars (compressed hydrogen), hence reducing the size of the hydrogen tanks. This solution has been already used in the development of the Toyota Mirai, which has 114kW/155hp power and a 500km range with a fuel consumption of 0.76 kg H2/100km^10^. Similarly, Honda Clarity could reach the range of 650km with 5kg of hydrogen tank capacity at the rated power of 130kW/ 176hp^11^. Although pressurizing the hydrogen is a feasible solution, it will demand further costs and safety procedures to reach the 700 bars. In other words, the best solution would be to reach the same driving range without pressurizing the hydrogen.

The other available hydrogen storage methods are^12^: liquefied hydrogen, cryo-compressed hydrogen, physically adsorbed hydrogen, metal hydrides, complex hydrides and liquid organic hydrides. By liquefied hydrogen^13^, the energy density will be equal to $8 \left(\frac{MJ}{L} H_2\right)$ and can be considered as an efficient method since it is also non-corrosive. However, the high cost of this method^14^, net heating value loss during the liquefaction process^15^, and the boil-off phenomenon^16^ are the barriers toward the commercialization of this method.

In this regard, Metal-Organic Framework (MOF) can be used to increase the hydrogen adsorption in the hydrogen tank due to higher gravimetric storage density. In this field, Chen et al.^17^ analyzed different MOFs and porous structures that improve the hydrogen storage capabilities. Gómez-Gualdrón et al.^18^ analyzed the trade-off between volumetric and gravimetric cryo-adsorbed hydrogen deliverable capacity and calculated that the maximum and minimum deliverable capacity in the MOF series of NU-1101, NU1102, and NU-1103 is ca. 40% gravimetrically, while only ca. 10% volumetrically. Inline with Gómez-Gualdrón et al.^18^, Yang et al.^19^ analyzed different hydrogen tanks filled with several MOFs and concluded that hydrogen stroage capability of cryo-compressed hydrogen storage alongside adsorption is higher than pure adsorption hydrogen storage. Xu et al.^20^ proposed the usage of Pd@MOF-808 as a solution to improve the hydrogen storage although instability was a barrier. Purewal et al.^21^ analyzed MOF-5, IRMOF-20, SNU-70, UMCM-9, DUT-23 (Co), and NU-100. The results indicated the promising results of MOF-5. Sridhar and Kaisare^22^ analyzed three hydrogen adsorption models of Unilan^23^, Modified Dubinin-Astakhov DA^24^, and Tóth^25^. The results indicated minor differences in the amount of velocity profiles. Suresh et al.^26^ suggested a method to modify the crystal morphologies of the MOFs to improve the hydrogen storage capabilities, while Jaramillo et al.^27^ proposed the usage of Vanadium (II)-dihydrogen as a solution. A review and comparison of the hydrogen tanks filled with MOFs, Multi-walled carbon nanotubes, and graphene were developed by Gangu et al.^28^, while Shet et al.^29^ specifically evaluated different types of MOFs for hydrogen storage improvements. Among different types of MOFs, the MOF-5 has shown promising results to increase the hydrogen storage up to wt. 10% absolute at 70 bar and 77K. It is believed that the low thermal conductivity of the MOF-5 can reduce the performance of the system when rapid gas uptake and release is required^30^. Although there have been studies to evaluate the overall possibilities of using MOF-5 to improve the adsorption of hydrogen in the hydrogen tanks, there is not a comprehensive study to simulate and characterize the changes in the hydrogen adsorption once the hydrogen tanks are filled with different types of MOFs.

### Novelties

The goal of this study is to use computational fluid dynamic methodologies to model a hydrogen tank filled with MOFs and to analyze the hydrogen adsorption by the changes in time. This study can be a step forward in improving the design of the hydrogen tanks, which will facilitate hydrogen storage at low pressures close to the ambient temperatures. This study can be also a good start to finding the right type of MOF to be used in the hydrogen tanks to have the highest possible hydrogen adsorption at the lowest required pressure possible. The developed model is based on mass, momentum, and energy conservation equations of the adsorbent-adsorbate system composed of gaseous and adsorbed hydrogen, adsorbent bed, and tank wall. It is noteworthy to mention that the adsorption process is based on the modified Dubinin-Astakov (D-A) adsorption isotherm model. A parametric study will be done to monitor the changes in the mass of adsorption, average temperature, and isosteric heat of adsorption during the transient adsorption of hydrogen. Although the characteristics of MOF-5 are known, there are many unknown MOFs that may have better hydrogen adsorption at lower pressures. Thus, twenty-seven simulations were done by the changes in the density, specific heat, and conductivity of the MOF to be used as a dataset to analyze 729220 different MOFs using an ANN model. The generated ANN model will be used to determine the optimum MOF, that is unknown at the moment, for hydrogen storage at low pressures. This study can be a valid reference for researchers in discovering novel MOFs for hydrogen storage applications.

Overall, the main novelties of this study are proposing and simulating (using computational fluid dynamics, CFD) the usage of MOFs to improve hydrogen storage in comparison to the conventional methods to increase the compression pressure to 700 bar to increase the hydrogen storage. Additionally, A detailed optimization was carried out using the Artificial Neural Network (ANN) modeling to obtain the optimized MOF considering the density, specific heat, and conductivity. The optimized MOF benefits from the highest adsorption while the required pressure to compress the hydrogen is the minimum.

## Methodology

**Figure 1:**
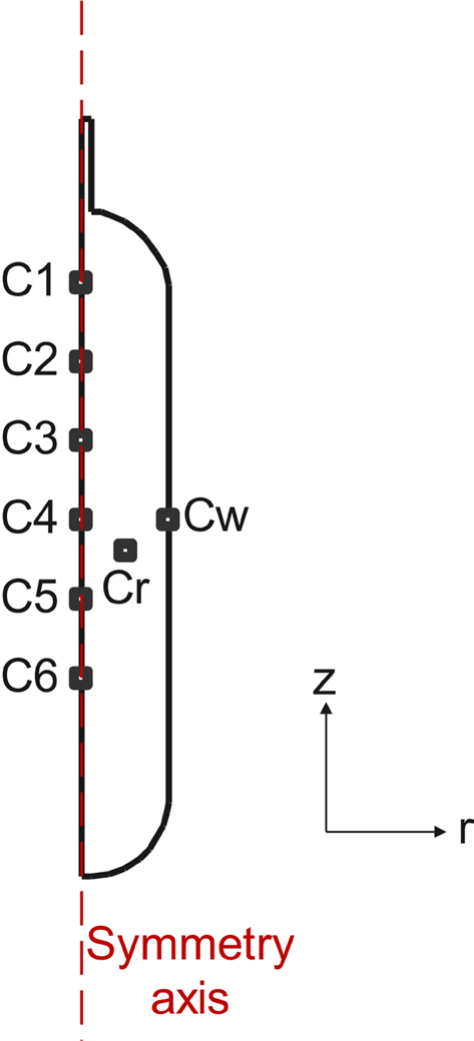
A schematic of the geometry of the considered tank to be analyzed in this study.

In this study, Computational Fluid Dynamic (CFD) methodologies have been used followed by the governing equations given in Section "Governing equations". The utilized geometry to perform the CFD studies is based on Fig. 1 and the simulations have been performed in the COMSOL commercial software. It should be noted that the simulations were carried out considering the axisymmetric condition on the z-axis, shown in Fig. 1. As can be seen in Fig. 1, considering the z-r coordination of the simulation domain, and the symmetry axis in the z-direction, the simulation was only done on the right side of the symmetry axis. The material of this hydrogen tank is considered to be stainless steel filled with MOF-5 adsorbents. The volume is 2.5 liters with the respective inner and outer radii of 4 mm and 5 mm at the entrance tank. In the middle of the tank, the inner and outer radii are 4.69 cm and 5.08 cm, respectively. To evaluate the differences in the pressure, temperature, and adsorption capabilities in the hydrogen tank, eight different points of C1, C2, C3, C4, C5, C6, Cr, and Cw are selected, as illustrated in Fig. 1, along the symmetry axis of the hydrogen storage tank. It should be noted that Cw and Cr are located close to the tank wall, and at the halfway to the tank wall, respectively.

The simulation has been performed considering the changes in the time with the time step of 1s. The selected material for the MOFs is the MOF-5 with the values of $n_{max}=151.8 \left(\frac{mol}{kg}\right)$, $p_0=1246 MPa$, $\alpha _{D-A}=1941 \frac{J}{mol}$, and $\beta _{D-A}=19.2 \left(\frac{J}{mol.K}\right)$ in the temperature range of 77K to 300K. The hydrogen is also assumed to be ideal gas in this study with the specific heat of $14700 \left(\frac{J}{kg.K}\right)$, conductivity of $0.206 \left(\frac{W}{m.K}\right)$, and the dynamic viscosity of $8.4e^{-6} (Pa.s)$. The tanks is made of steel with the bulk density of $7830 \left(\frac{kg}{m^3}\right)$, specific heat of $468 \left(\frac{J}{kg.K}\right)$, and the conductivity of $13 \left(\frac{W}{m.K}\right)$. The MOF-5 adsorbents have also the bulk density of $130 \left(\frac{kg}{m^3}\right)$, the specific heat of $780 \left(\frac{J}{kg.K}\right)$, the conductivity of $0.088 \left(\frac{W}{m.K}\right)$, the bed porosity of 0.246, and the particle diameter of 0.36*mm*.

It is noteworthy to mention that, for certain types of mathematical problems, the convergence conditions by Courant-Friedrich-Lewy should be met to reach the convergence of the partial differential equations (PDEs). In other words, whenever the explicit time integration schemes are being used to solve PDEs, the Courant number should be presented and evaluated for the convergence of the equations to prevent the instability of the time-marching computer simulations. However, using the implicit time integration schemes, the Courant number does not need to be analyzed since there is not any sensitivity to define small time steps to satisfy the Courant conditions. Moreover, the Variable-steps Solvers will be able to handle any instability condition since it would minimize the time step dynamically whenever large gradients appear.

### Boundary conditions

Considering the boundary conditions, the hydrogen tank has the initial pressure of 0.03208*MPa* and wall temperature of 302*K*, while having the heat transfer constant of $36 \left(\frac{W}{m^2.K}\right)$ between the steel wall and the water temperature. The thermodynamic properties of the hydrogen flow at the entrance and exit are calculated using the NIST refprop^31^ in the MATLAB software. Table 1 also presents the selected mass flow rates and hydrogen temperatures for different selected periods of time.

**Table 1:** The selected input boundary conditions of the hydrogen flow to the tank as a function of time to perform the CFD simulation.

Time (s)	Mass flow rate $\left(\frac{kg}{s}\right)$	Mass flux $\left(\frac{kg}{m^2.s}\right)$	Hydrogen temperature (K)
0-953	2.05$e^{-5}$	0.41	301.7
953-3822	0	0	302.5
3822-4694	– 2.19$e^{-5}$	– 0.44	297.7
4694-6000	0	0	298.6

## Governing equations

### Distributed parameter model

Assuming the density of the hydrogen gas as $\rho _g \left(\frac{kg}{m^3}\right)$, and the bed porosity as $\varepsilon _b$, the formulation of the mass conservation can be presented as follows:

1 $$\begin{aligned} \frac{\partial (\varepsilon _b \rho _g)}{\partial t}+\nabla .(\rho _g \bar{v})=S_m, \end{aligned}$$

where, $S_m (\frac{kg}{m^3.s})$ is the mass source term, which is a function of the adsorption (see Eq. (2)), and $\bar{v} (\frac{m}{s})$ is the Darcy velocity vector:

2 $$\begin{aligned} S_m=-\frac{\partial }{\partial t}(\rho _b q_a)=-(1-\varepsilon _b)\rho _p M_{H_2} \frac{\partial n_a}{\partial t}, \end{aligned}$$

where, $\rho _b \left(\frac{kg}{m^3}\right)$ is the bed density and can be calculated by $\rho _b=(1-\varepsilon _b)\rho _p$, while $q_a (\frac{kg}{kg})$ is the ratio of the adsorbate to the adsorbent considering the absolute adsorption amount, $n_a (\frac{mol}{kg})$ and the molecular mass of hydrogen, $M_{H_2} (\frac{kg}{mol})$.

To calculate the Darcy velocity, the momentum conservation will be used as follows:

3 $$\begin{aligned} \bar{v}=-\frac{\kappa }{\mu }\nabla p, \end{aligned}$$

where, $\kappa (m^2)$ is the permeability (see Eq. 4), and $\mu (Pa.s)$ is the dynamic viscosity.

4 $$\begin{aligned} \kappa =\frac{D_{p}^{2} \varepsilon _{b}^{3}}{150(1-\varepsilon _b)^2}. \end{aligned}$$

Here, $D_p (m)$ is the diameter of the adsorbent nano-particles. Furthermore, Eq. 5 takes into account the energy conservation:

5 $$\begin{aligned} (\rho c_p)_{eff} \frac{\partial T}{\partial t}+ \rho _g c_{pg} \bar{v}. \nabla T=\nabla . (k_{eff} \nabla T)+Q+W+\phi, \end{aligned}$$

where, $Q (\frac{W}{m^3})$ is the adsorption heat, $W (\frac{W}{m^3})$ is the required work in the mentioned process, $\phi (\frac{W}{m^3})$ is the viscous dissipation, *T*(*K*) is the temperature, $k_{eff} (\frac{W}{m.K})$ is the effective thermal conductivity of activated carbon bed (see Eq. 6), $c_p (\frac{J}{kg.K})$ is the specific heat of the adsorbent particles, and $c_{pg} (\frac{J}{kg.K})$ is the specific heat capacity of the hydrogen gas.

6 $$\begin{aligned} k_{eff}=\varepsilon _b k_g +(1-\varepsilon _b)k_s. \end{aligned}$$

Here, $k_s (\frac{W}{m.K})$ is the thermal conductivity of the adsorbent, and $k_g (\frac{W}{m.K})$ is the thermal conductivity of the hydrogen gas. Using Eq. 7, the share of adsorbed hydrogen to the effective heat capacity will be obtained:

7 $$\begin{aligned} (\rho c_p)_{eff}=\varepsilon _b \rho _g c_{pg}+(1-\varepsilon _b) \rho _p (c_{ps}+q_a c_{pa}), \end{aligned}$$

where, $c_{ps} (\frac{J}{kg.K})$ is the specific heat capacity of the adsorbent. Simplifying Eq. (7) results in Eq. (8) assuming that the adsorbed hydrogen gas higher than the critical temperature is almost equal to the compressed gas, i.e. $c_{pa}=c_{pg}$:

8 $$\begin{aligned} (\rho c_p)_{eff}=\varepsilon _b \rho _g c_{pg}+\rho _b n_a M_{H_2} c_{pa}+\rho _b c_{ps}. \end{aligned}$$

Equations (9), (10) and (11) will also show the parameters *Q*, *W*, and $\phi$, respectively:

9 $$\begin{aligned}{} & {} Q=-\frac{S_m \Delta H}{M_{H_2}}=\rho _b \Delta H \frac{\partial n_a}{\partial t}, \end{aligned}$$

10 $$\begin{aligned}{} & {} W=\varepsilon _b \beta _T T \frac{Dp}{Dt}=\varepsilon _b \beta _T T\left(\frac{\partial p}{\partial t}+\bar{u}.\nabla p\right)=\beta _T T \left(\varepsilon _b \frac{\partial p}{\partial t}+\bar{v}.\nabla p\right), \end{aligned}$$

11 $$\begin{aligned}{} & {} \phi =\tau :\nabla \bar{v}=\mu \left[\nabla \bar{v}+(\nabla \bar{v})^T-\frac{2}{3}(\nabla .\bar{v})I\right]:\nabla \bar{v}, \end{aligned}$$

where, $\Delta H \left(\frac{J}{mol}\right)$ is the isosteric heat of adsorption, while $\bar{u} (\frac{m}{s})$ and $\bar{v}=\varepsilon _b \bar{u} \left(\frac{m}{s}\right)$ are the velocity in the porous channels and the Darcy velocity through the porous regions, respectively. *I* is the unit tensor, $\beta _T (\frac{1}{K})$ is the volume expansion coefficient, and $\tau (Pa)$ is the shear stress.

#### Lumped parameter model

Based on the mass conservation, the input and output flow of hydrogen to the hydrogen tanks is equal as follows:

12 $$\begin{aligned} \frac{dm_t}{dt}=\dot{m}_i-\dot{m}_e, \end{aligned}$$

where, $m_t$, $\dot{m}_i$, and $\dot{m}_e$ indicate the total mass in the tank, the mass flow rate to the tank, and the mass flow rate out of the tank, respectively.

Considering the conservation of mass and the ideal gas equation, the pressure can be calculated:

13 $$\begin{aligned} p=\frac{m_g ZR_uT}{M_{H_2}V_g}, \end{aligned}$$

where, $R_u$ is the universal gas constant and *Z* is the gas compression factor. To perform the comparison between the obtained temperatures from the lumped and distributed parameter models, a thermal average temperature is calculated as of Eq. (14):

14 $$\begin{aligned} \bar{T}_C=\frac{\int _V (\rho c_p)_{eff}TdV}{\int _V (\rho c_p)_{eff}dV}, \end{aligned}$$

where, the differential volume is $dV=2\pi r dr dz$.

#### Adsorption model

The developed adsorption model is based on the modified Dubinin-Astakhov (D-A)^32^ following Eq. (15) for the absolute adsorption:

15 $$\begin{aligned} n_a=n_{max} exp\left[-\left(\frac{R_u T}{\alpha _{D-A}+\beta _{D-A}T}ln\frac{p_0}{p}\right)^m\right], \end{aligned}$$

where, $\alpha _{D-A} \left(\frac{J}{mol}\right)$ and $\beta _{D-A} \left(\frac{J}{mol.K}\right)$ are the enthalpic and entropic factors, respectively. The coefficient *m* can be varied based on the selected MOF and $m=2$ for the adsorption of MOF-5 and activated carbon. The parameters, $n_{max}$, $p_0$, $\alpha _{D-A}$, and $\beta _{D-A}$ are selected from Refs.^32–34^. Using the chain-rule, the absolute adsorption can be obtained using Eq. (16):

16 $$\begin{aligned} \frac{\partial n_a}{\partial t}=m n_a \left(\frac{R_u T}{\alpha _{D-A}+\beta _{D-A}T}ln\frac{p_0}{p}\right)^m\left[\frac{1}{ln\left(\frac{p_0}{p}\right)}\frac{\partial p}{p\partial t}-\frac{\alpha _{D-A}}{\alpha _{D-A}+\beta _{D-A}T}\frac{\partial T}{T\partial t}\right]. \end{aligned}$$

Based on the D-A model^32^, the isosteric heat of adsorption can be obtained using Eq. (17) when $m=2$:

17 $$\begin{aligned} \Delta H=\alpha _{D-A}\sqrt{ln\left(\frac{n_{max}}{n_a}\right)}. \end{aligned}$$

### Artificial Neural Network (ANN) modeling

**Figure 2:**
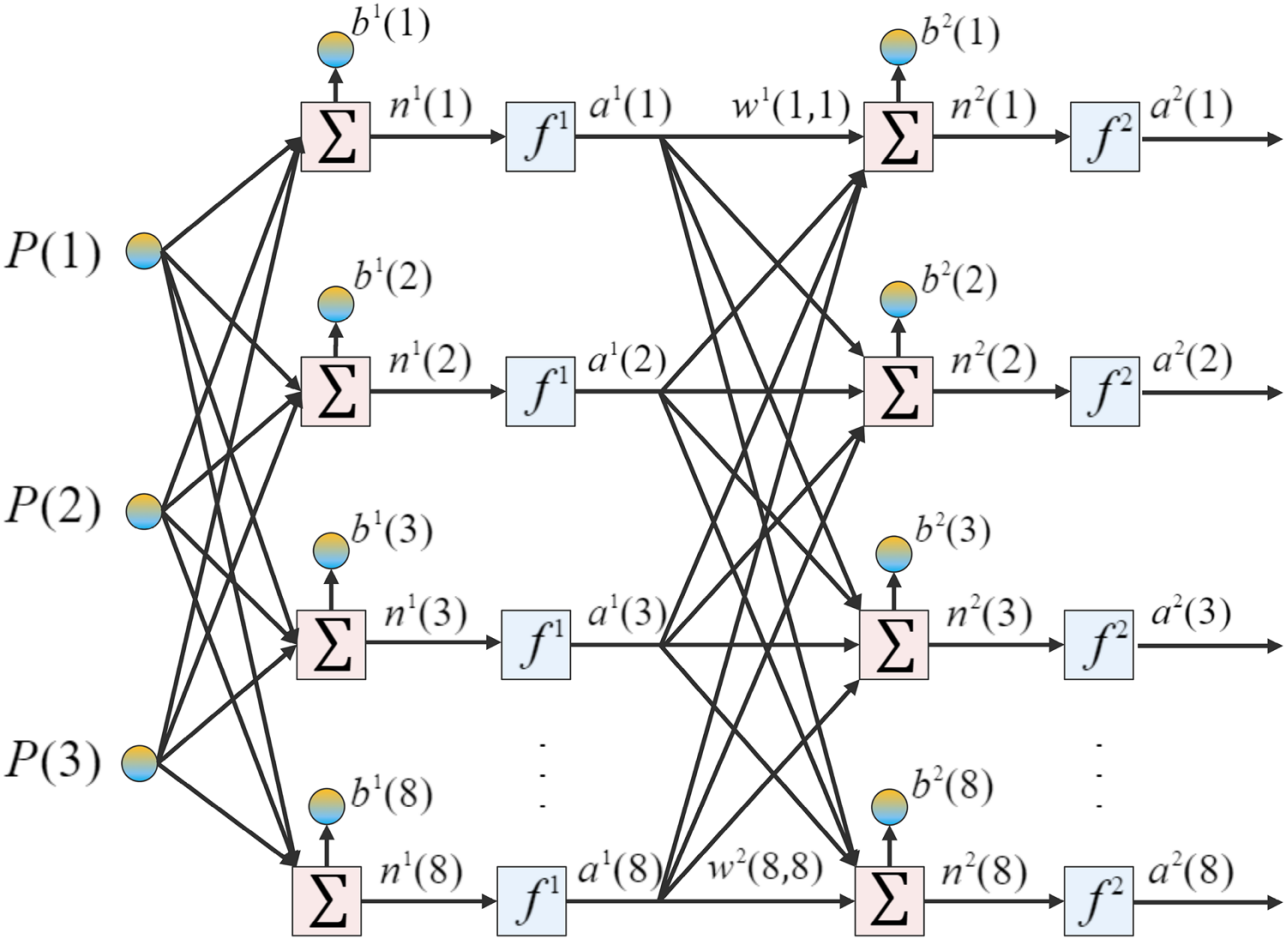
A schematic of the eight-layered feed-forward ANN model.

The utilized algorithm for developing and training the ANN model is the Levenberg-Marquardt algorithm^35^ based on the 27 observations given by Table 2 with three input parameters of the bulk density, specific heat, and conductivity. Figure 2 also shows a schematic of the eight-layered feed-forward ANN model used in this study.

The net inputs and outputs to the unit *i* can be demonstrated by Eqs. (18) and (19):

18 $$\begin{aligned}{} & {} n^{k+1}(i)=\Sigma ^{S_k}_{j+1}\omega ^{k+1}(i,j)a^{k}(j)+b^{k+1}(i), \end{aligned}$$

19 $$\begin{aligned}{} & {} a^{k+1}(i)=f^{k+1}(n^{k+1}(i)). \end{aligned}$$

Considering an eight-layered ANN model, the matrix of the equations can be shown by Eqs. (20) and (21):

20 $$\begin{aligned}{} & {} a^0=P \end{aligned}$$

21 $$\begin{aligned}{} & {} a^{k+1}=f^{k+1}(\omega ^{k+1}a^{k}+b^{k+1}),k=0,..., 7 \end{aligned}$$

Further details on the utilized ANN model and the governing equations can be found in the previous contribution of the authors in Ref.^36^.

## Results

**Figure 3:**
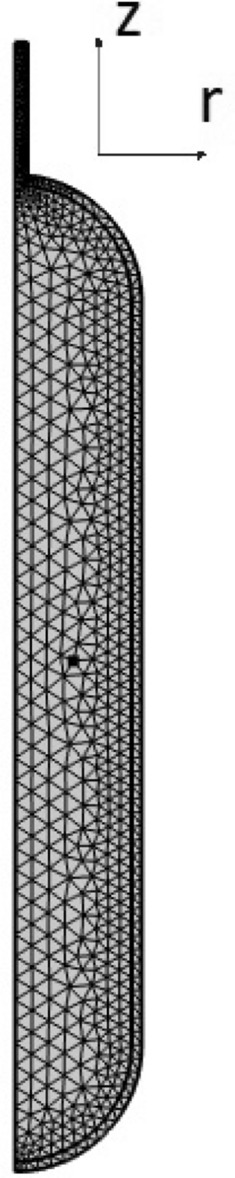
The considered extra fine mesh to perform the simulations.

To perform the simulation studies, the developed geometric model in Fig. 1 meshed in the Free Mesher module of the COMSOL software. Figure 3 shows the details of this mesh for the considered geometry.

**Figure 4:**
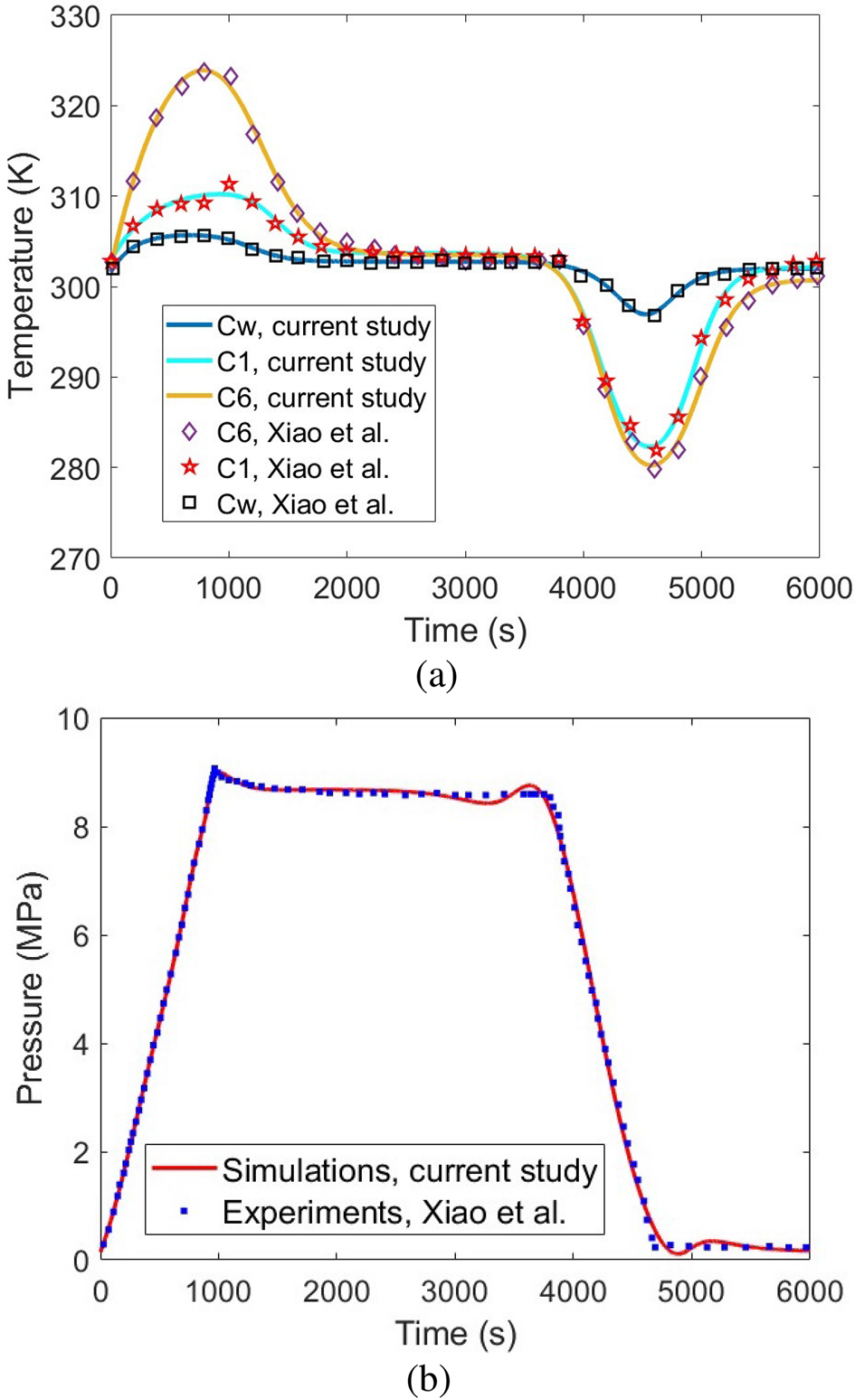
Model validation of the simulation results of the current study with the reported temperature and pressure by the experimental setup, developed by Xiao et al. at the Hydrogen Research Institute, University of Quebec at Trois-Rivieres^30,37,38^: (**a**) Temperature validation considering Fig. 1 for points C1, C6, and Cw, (**b**) Pressure validation.

Model validation has been done using the reported data by the Hydrogen Research Institute, University of Quebec at Trois-Rivieres^30,37,38^ based on Test No. 20. Figure 4 shows the comparison between the obtained simulation data and the reported data by Xiao et al., Refs.^30,37,38^. As can be seen, the developed simulation model has high precision/accuracy to capture similar results of the experimental setup in a transient simulation.

**Figure 5:**
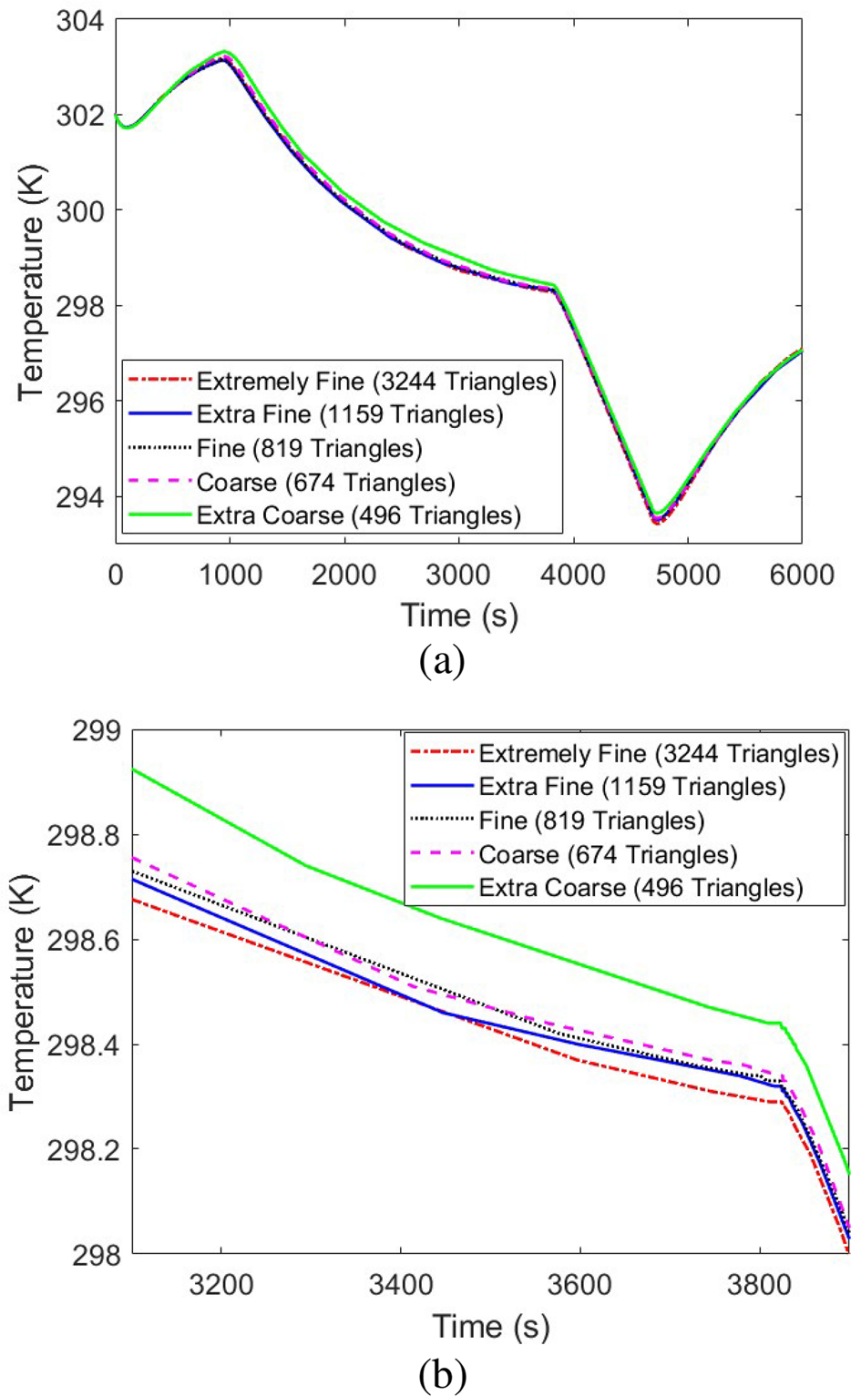
The grid independence study of the developed simulation model using the changes in the reported temperature at point Cw based on Fig. 1 by the variation of the number of existing triangles in the computational domain: (**a**) The whole simulation time, (**b**) Shorter simulation time to better visualize the impacts of changing the grid size.

**Figure 6:**
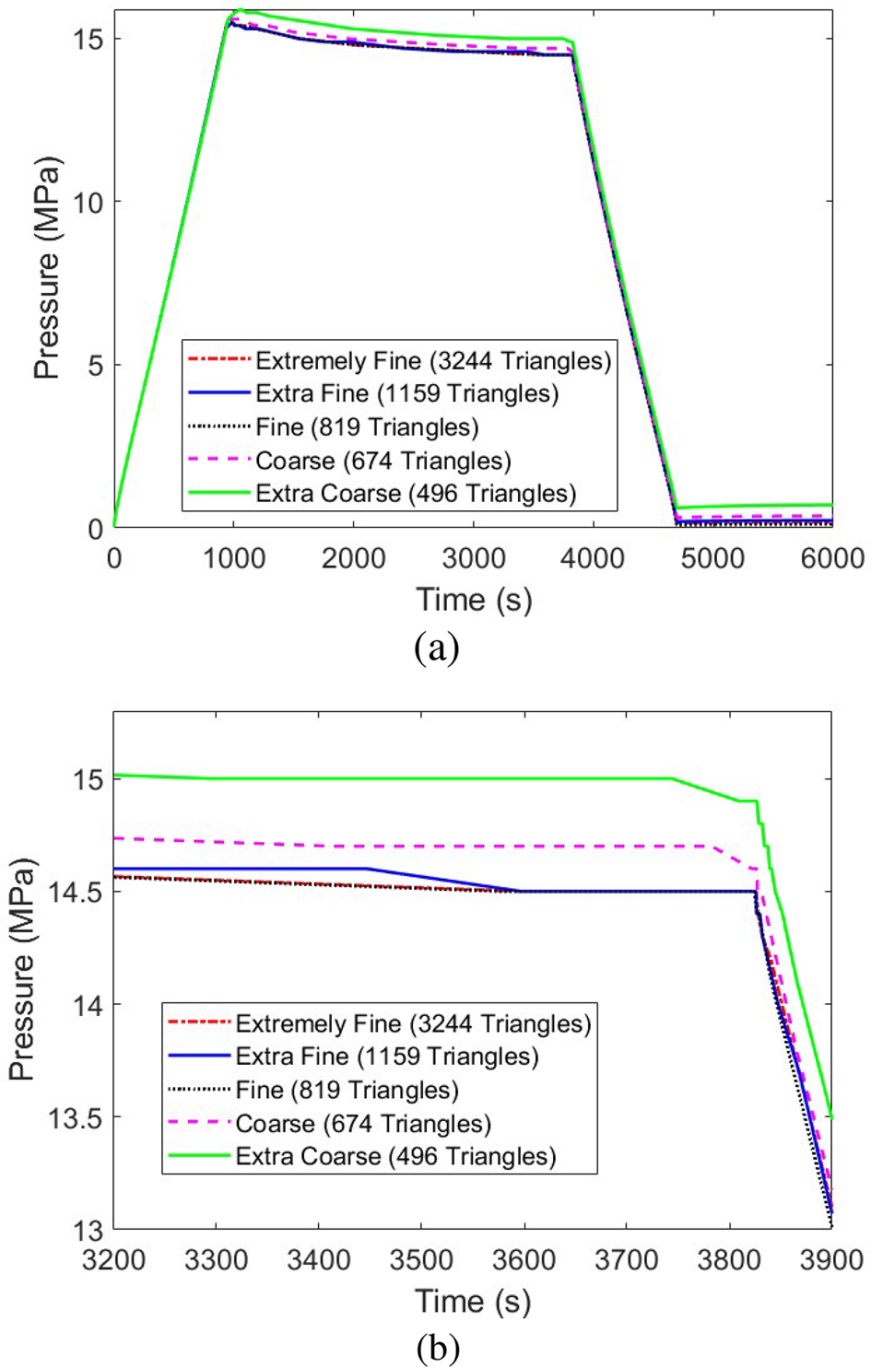
The grid independence study of the developed simulation model using the changes in the reported pressure by the variation of the number of the existing triangles in the computational domain: (**a**) The whole simulation time, (**b**) Shorter simulation time to better visualize the impacts of changing the grid size.

After model validation, a detailed grid independence study should be developed to verify the independence of the obtained results from the mesh size in the computational model. In this regard, the temperature at point $C_w$ based on Fig. 1 and the pressure were selected to be analyzed in five different numbers of triangles in the computational domain, namely, Extremely Fine with 3244 triangles, Extra Fine with 1159 triangles, Fine with 819 triangles, Coarse with 674 triangles, and Extra Coarse with 496 triangles. Performing the grid independence study, Fig. 5 shows the corresponding results for the temperature at point $C_w$ based on Fig. 1, while Fig. 6 illustrates that of the average pressure in the tank. Considering the obtained results, “Extra Fine” mesh quality with 1159 triangles showed promising results with acceptable computational time, so was selected for the rest of the study.

Considering the time-step independence study, it should be noted that the COMSOL software uses a “Variable-step solver” for transient problems. The variable-step solver changes the step size during the simulation and reduces the step size to increase accuracy when model states are changed rapidly. The solver also increases the step size to avoid taking unnecessary steps when model steps are changed slowly. As with fixed-step solvers, the set of variable-step solvers comprises a discrete and a collection of continuous solvers. Unlike the fixed-step solvers, the step size varies dynamically based on the local error. In other words, in the fixed-step solvers, as a fixed time-step is being used, a grid independence study for the time step is needed to verify the results, however, in variable-step solvers, the time step is being changed continuously between the order of magnitude of 1e – 4 to 1 second to obtain the most accurate/precise results, hence the study is numerically independent of the time step.

**Figure 7:**
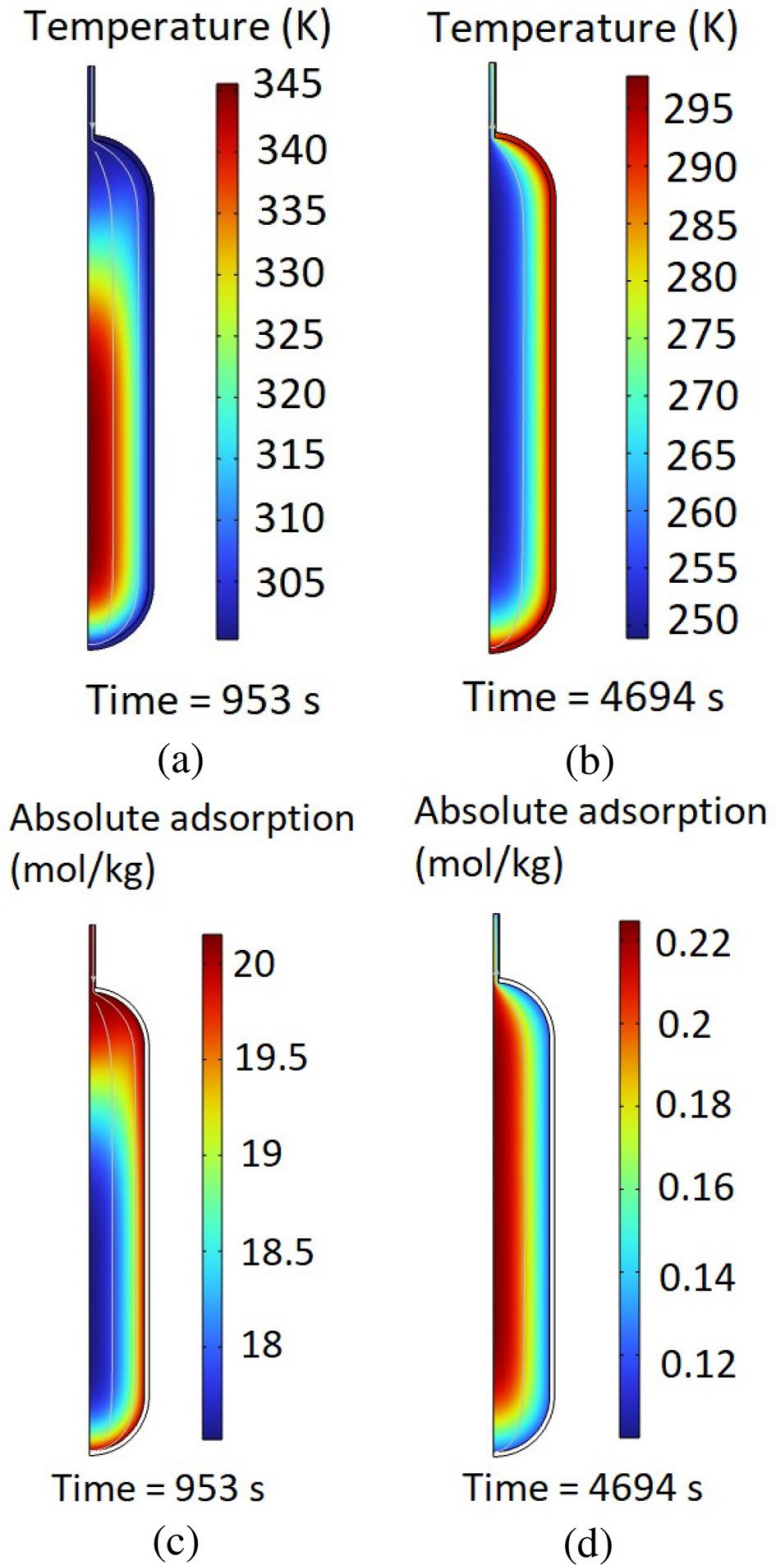
The obtained contours of the distribution of the temperature and absolute adsorption in the hydrogen tanks by the changes in the time: (**a**) The contours of temperature at the time steps of 953s, (**b**) The contours of temperature at the time steps of 4694s, (**c**) The contours of absolute adsorption at the time steps of 953s, (**d**) The contours of absolute adsorption at the time steps of 4694s.

Once the validation and the grid independence study of the simulation model are performed, the CFD analysis can be done using the developed model. This study considers a transient, time-dependent, simulation of the absolute adsorption and temperature variation throughout the hydrogen tank. In this regard, the contours of the temperature distribution and the absolute adsorption distribution at the time steps of 953s and 4694s are obtained and illustrated in Fig. 7. As can be seen in Fig. 7a and b, at the beginning of the hydrogen storage process, the temperature is higher in the center of the tank in comparison to the walls. However, with passing of the time, although the whole temperature will reduce throughout the tank, there is an increased temperature at the walls. The reason is the lower thermal conductivity of the MOF-5, which has filled the inner section of the tank, in comparison to the stainless steel, which is considered as the material of the tank’s walls. Additionally, the coolant is located on the outside of the wall, hence when the inner temperature of the tank is higher than the temperature of the coolant, Fig. 7a, there will be heat transfer from the tank to the coolant, leading to lower temperatures at the wall. However, once the inner temperature of the tank is lower than the temperature of the coolant, Fig. 7b, the direction of the heat transfer will be from the coolant to the tank which results in higher temperatures at the wall. Considering the contours of the absolute adsorption, which are shown in Fig. 7c and d, higher temperatures result in lower absolute adsorption. In other words, the absolute adsorption of hydrogen using the MOF-5 in the hydrogen storage tanks has an opposite relationship with the temperature which leads to lower values of the absolute adsorption once the temperature is high.

**Figure 8:**
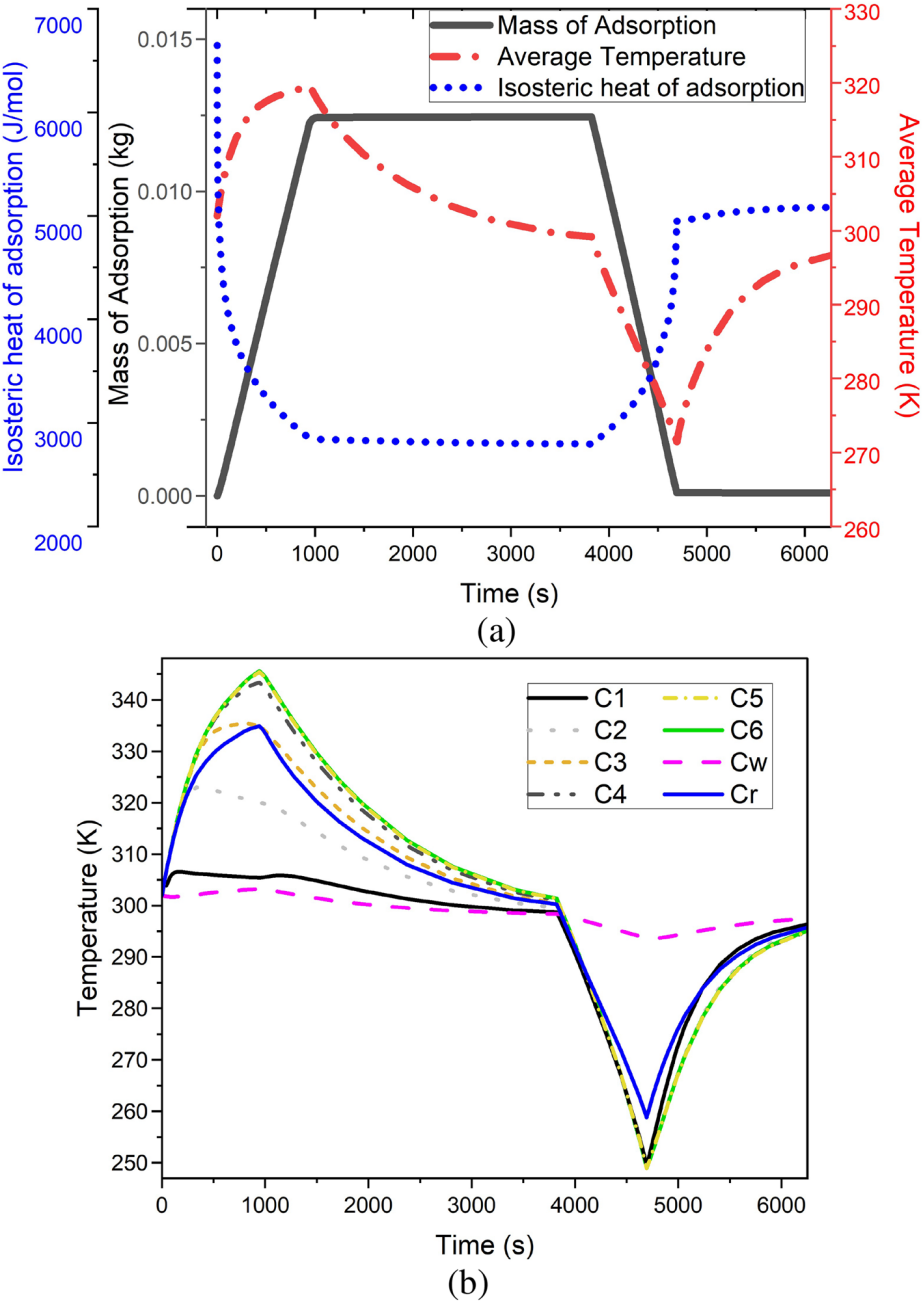
The characteristics of the simulated hydrogen tank filled with MOF-5 by the changes in the time: (**a**) The changes in the mass of adsorbed hydrogen, the time-dependent thermal average temperature, and the isosteric heat of adsorption, (**b**) The time-dependent variations of the temperature in the selected points based on Fig. 1.

Regarding the storage process of the hydrogen in the tank, Fig. 8a shows the mass of the adsorbed hydrogen by the changes in time. As can be seen, the storage process takes around 1000s to completely fill the tank and reach the adsorbed hydrogen value of $1.23e-2$. Figure 8b illustrates the changes in the temperatures of eight selected points based on Fig. 1 throughout the tank. During the storing process, the temperatures of the points that are more in the center of the tank have higher values than those that are close to the wall. Additionally, the temperature at the selected point of *Cw* has the least changes during the storing and discharging processes during the period of 6000s. Figure 8a gives more detail about the temperature variations during the selected time period by presenting the thermal average temperature in the whole hydrogen tank, which is filled with MOF-5. Figure 8b also presents the time-dependent variations of the temperature in the selected points based on Fig. 1. Considering Eq. (17), the isosteric heat of adsorption can be calculated in the simulated hydrogen tank at point C4. Once the storing process of the hydrogen is done, the quantity of the stored hydrogen in the tank will be enhanced by the passing of time, which results in the slow mitigation of the isosteric heat of adsorption. From around 1000 s to 4000 s, as the absolute adsorption is not changing and no more hydrogen is trying to be stored, the value of the isosteric heat of adsorption will remain constant as well. However, the corresponding value of the isosteric heat of adsorption experiences a smooth escalation during the discharging process of the adsorbed hydrogen so that the tank will be empty.

### Artificial neural network (ANN) modeling

The research on Metal Organic Frameworks (MOFs) have revealed that although the properties of many MOFs are available, many other MOFs are not discovered yet. Concentrated efforts have been made to synthesize and discover novel MOFs for different applications including hydrogen storage but in some cases, even the desired chemical/mechanical properties are not known. Thus, it will be valuable to dedicate investigations to figure out the desired values of the optimal MOF for hydrogen storage. This study uses CFD simulations to build up a database for training an ANN model to analyze 729220 different MOFs that are similar to MOF-5 except for the values for the density, conductivity, and specific heat. Once the ANN model is developed the mass of adsorption and the required pressure for hydrogen adsorption will be determined to find the optimum MOF for hydrogen storage. Table 2 shows the results of CFD simulations for twenty-seven different cases of the densities, conductivities, and specific heats. Regarding Table 2, as the MOF characteristics have great influence on the outputs parameters, three main parameters of MOFs namely, density, specific heat, and conductivity were chosen to vary in their own range. For each parameter, three different values were considered and the outputs of the system, which are hydrogen adsorption, average temperature, and average pressure were determined for each case. Then, the obtained dataset of Table 2 was used to train an artificial neural network including dedicated training, validating, and testing datasets.

**Table 2:** The performed simulations to obtain the pressure, average temperature, and the adsorption capability of MOF-4 in different conductivities, specific heats, and bulk densities.

Simulation number	Bulk density $\left(\frac{kg}{m^3}\right)$	Specific heat ($\frac{J}{kg.K}$)	Conductivity ($\frac{W}{m.K}$)	Hydrogen adsorption (g) at 1000 s	Average temperature (K) 1000 s	Pressure at at 1000 s (bar)
1	100	700	0.06	9.80	317.02	143
2	100	700	0.5	9.83	308.84	140
3	100	700	1	9.90	306.43	139
4	100	775	0.06	9.80	316.45	143
5	100	775	0.5	9.84	308.84	140
6	100	775	1	9.90	306.46	139
7	100	850	0.06	9.80	316.15	143
8	100	850	0.5	9.84	308.84	140
9	100	850	1	9.90	306.48	139
10	200	700	0.06	7.07	316.15	102
11	200	700	0.5	7.14	309.8	102
12	200	700	1	7.10	306.68	98.7
13	200	775	0.06	7.10	315.41	102
14	200	775	0.5	7.14	309.69	101
15	200	775	1	7.1	307.15	98.9
16	200	850	0.06	7.07	314.93	102
17	200	850	0.5	7.14	309.57	101
18	200	850	1	7.10	306.68	98.8
19	300	700	0.06	5.56	314.35	80.4
20	300	700	0.5	5.57	309.28	78.8
21	300	700	1	5.57	307.17	78.1
22	300	775	0.06	5.50	313.98	79.5
23	300	775	0.5	5.57	309.12	78.7
24	300	775	1	5.57	307.12	78.1
25	300	850	0.06	5.50	313.44	79.3
26	300	850	0.5	5.57	308.95	78.7
27	300	850	1	5.57	307.06	78.1

**Figure 9:**
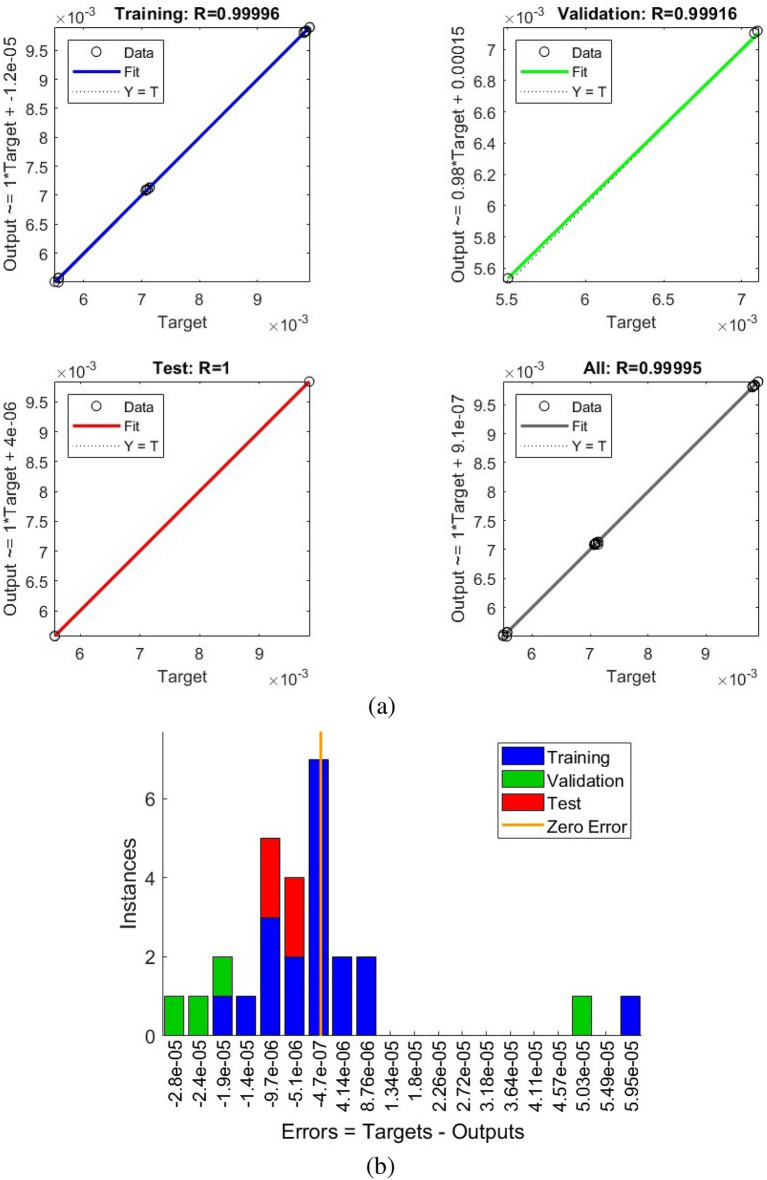
The regression and the error histogram diagrams of the trained and simulated data using the ANN model and the COMSOL simulation model, respectively: (**a**) the regression diagram, (**b**) the error histogram diagram.

The simulated values in using the CFD methods will then be used to train an ANN model by dedicating 19 observations to training, 4 observations to validation, and 4 observations to testing. The mean squared error (MSE) was used to analyze the precision of the trained ANN model. The MSE values for the training, validation, and testing datasets were $2.65\times 10^{-10}$, $1.04\times 10^{-09}$, and $4.82\times 10^{-11}$, respectively. The regression diagram and the error histogram of the ANN model are also shown in Fig. 9. The training algorithm was Levenberg-Marquardt, while the number of layers (the number of neurons) for this ANN model is considered to be eight.

**Figure 10:**
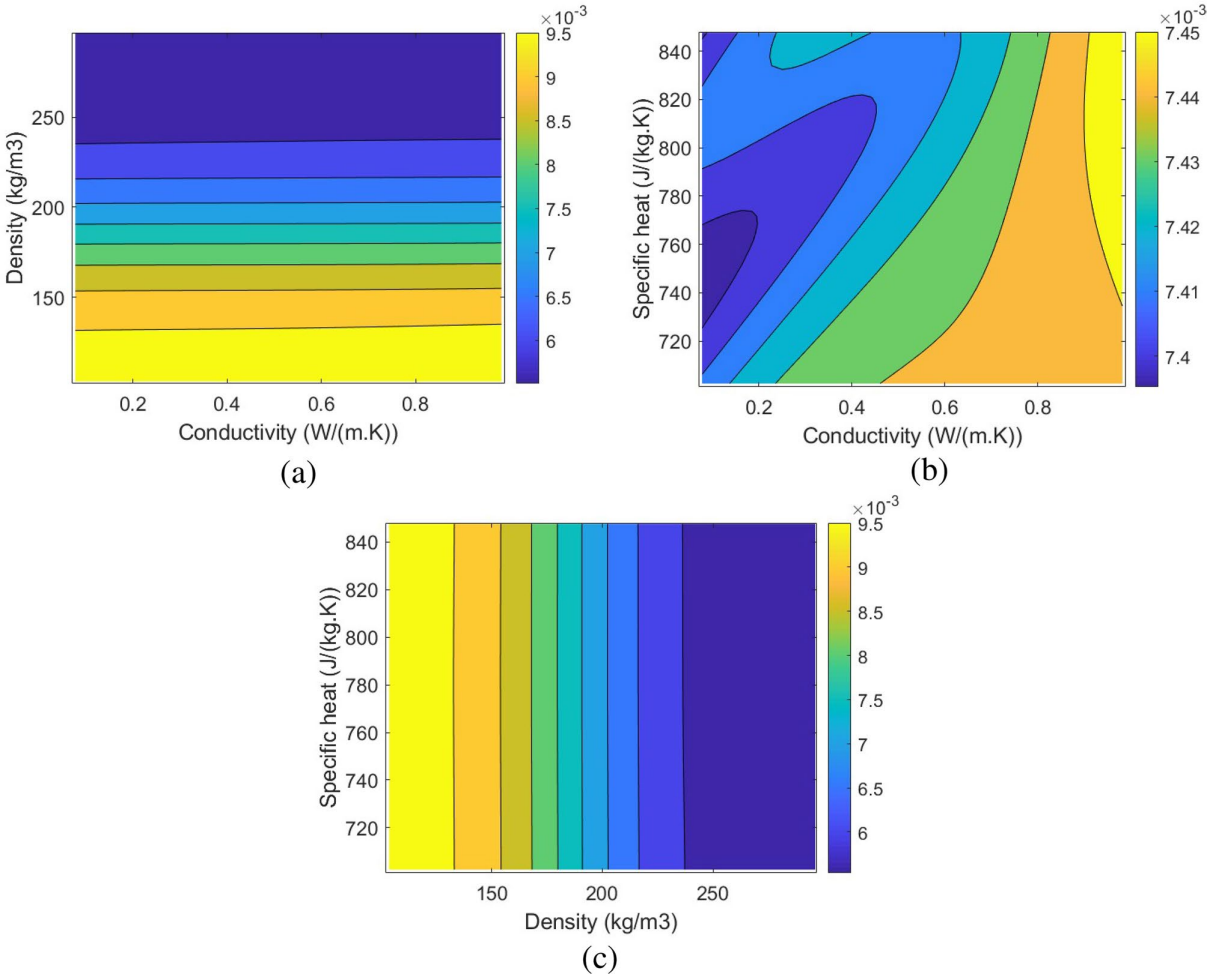
The changes in the Adsorption using the developed ANN model by the variation of the Conductivity and the density of the selected MOF: (**a**) 2D contour of the changes in the conductivity and the density, (**b**) 2D contour of the changes in the conductivity and the specific heat, (c) 2D contour of the changes in the specific heat and the density.

**Figure 11:**
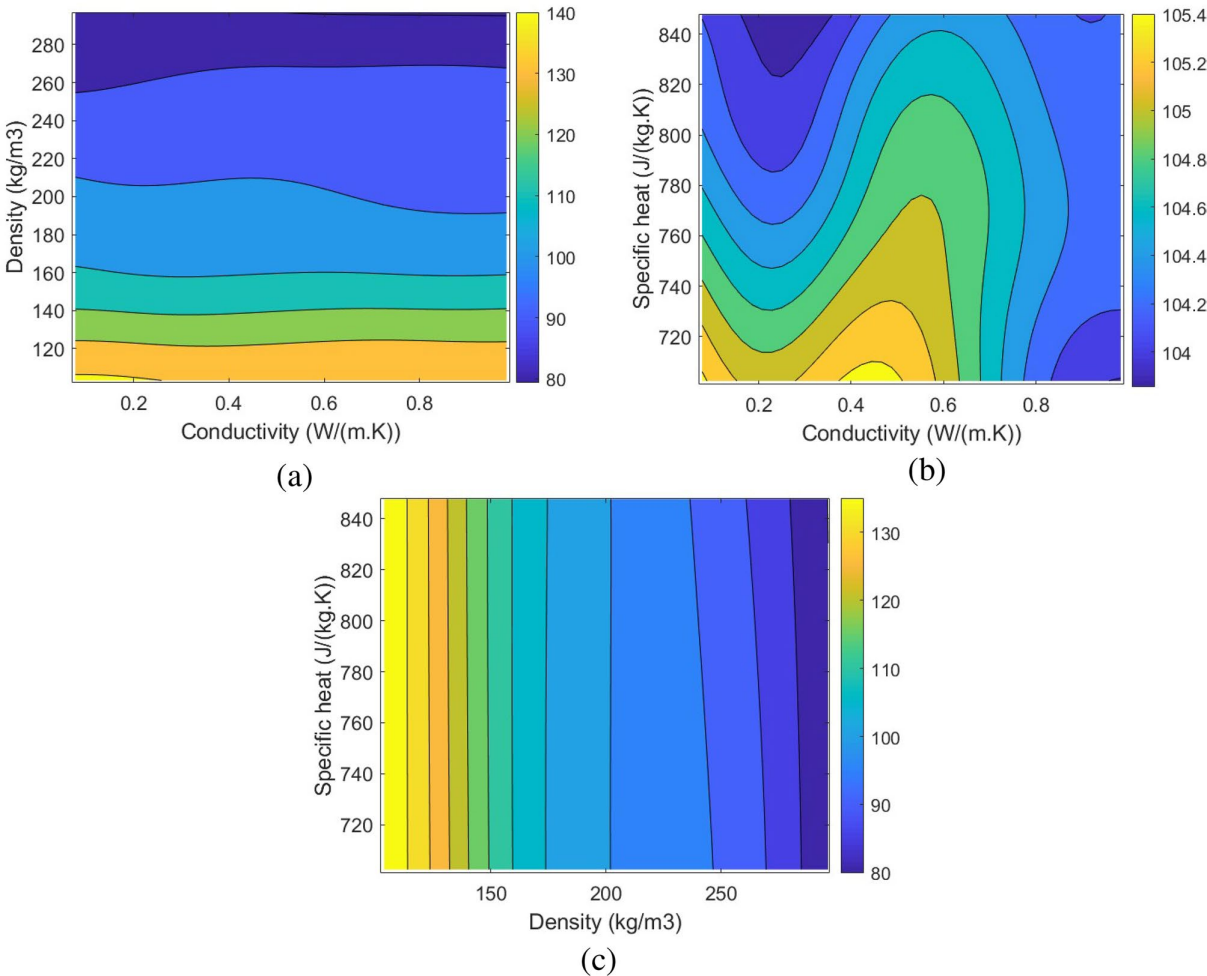
The changes in the pressure using the developed ANN model by the variation of the Conductivity and the density of the selected MOF: (**a**) 2D contour of the changes in the conductivity and the density, (**b**) 2D contour of the changes in the conductivity and the specific heat, (**c**) 2D contour of the changes in the specific heat and the density.

**Figure 12:**
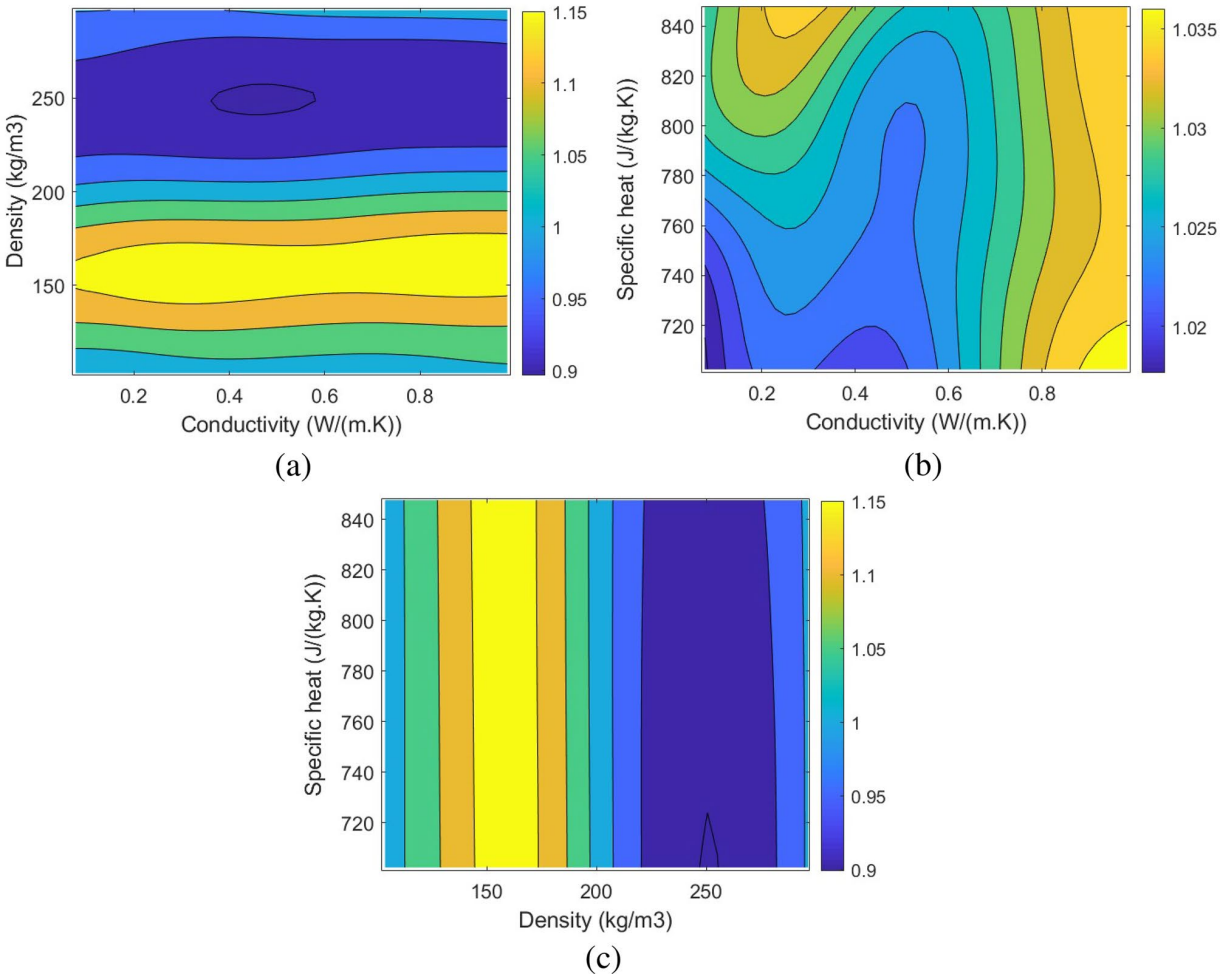
The changes in the Pressure Adsorption Parameter (PAP) using the developed ANN model by the variation of the Conductivity and the density of the selected MOF: (**a**) 2D contour of the changes in the conductivity and the density, (**b**) 2D contour of the changes in the conductivity and the specific heat, (**c**) 2D contour of the changes in the specific heat and the density.

Once the ANN model is trained, validated, and tested, it is possible to develop and analyze the changes in the mass of adsorption, the required pressure for hydrogen storage, and the Pressure Adsorption Parameter (PAP). PAP (Eq. (22)) is the new dimensionless parameter defined in this study to find the highest mass of adsorption while the required pressure for hydrogen storage is at the lowest. Figures 10, 11 and 12 show the corresponding contours of the variation in the mass of adsorption, required pressure for hydrogen storage, and PAP by the changes in the density, specific heat, and the conductivity of the possible MOF.

22 $$\begin{aligned} PAP=\frac{\frac{(Adsorption)_{new}}{(Adsorption)_{base}}}{\left(\frac{(Pressure)_{new}}{(Pressure)_{base}}\right)^{0.25}}. \end{aligned}$$

Using the developed ANN model based on the CFD simulations enabled the determination of the optimum MOF for hydrogen storage applications to have the highest mass of adsorption while benefiting from low storage pressures. The parameter PAP also enabled the single-objective optimization in a manner that the highest PAP will result in the most optimal condition for a MOF to be discovered. The results of the optimization revealed that the optimum MOF, which is unknown at the moment, should have a density of 100 $(kg/m^3)$, a conductivity of 0.7 (*W*/(*m*.*K*)), and a specific heat of 786 (*J*/(*kg*.*K*)) to reach the mass of adsorption of 0.0099 kg in 139 bar, which quite lower than 700 bar in conventional methods. Table 3 shows the details about the results of the single-objective optimization. The results of this study can be a valid reference for researchers who are active in discovering novel MOFs to find the right MOF for hydrogen storage applications.

**Table 3:** The optimum conditions of the adsorption and Pressure Adsorption Parameter (PAP) using the developed ANN model.

Parameter	Value
Optimization criterion	Maximum PAP
Maximum PAP	1.0384
Maximum adsorption	0.0099 (kg)
Minimum pressure	139 bar
Density	100 ($\frac{kg}{m^3}$)
Conductivity	0.7 ($\frac{W}{m.K}$)
Specific heat	776 ($\frac{J}{kg.K}$)

## Data Availability

The datasets used and/or analysed during the current study available from the corresponding author on reasonable request.
